# The Frequency of V122I Transthyretin Mutation in a Cohort of African American Individuals With Bilateral Carpal Tunnel Syndrome

**DOI:** 10.3389/fneur.2022.949401

**Published:** 2022-07-26

**Authors:** Jeffrey Z. Shije, Maria A. B. Bautista, Carmen Smotherman

**Affiliations:** ^1^Department of Neurology, University of Virginia School of Medicine, Charlottesville, VA, United States; ^2^Department of Neurology, University of Florida College of Medicine, Jacksonville, FL, United States; ^3^Department of Epidemiology, University of Florida College of Medicine, Jacksonville, FL, United States

**Keywords:** hereditary transthyretin amyloidosis (hATTR), V122I *TTR* mutation, carpal tunnel syndrome, African American health, amyloid neuropathies

## Abstract

**Introduction:**

Hereditary transthyretin amyloidosis (hATTR) can cause multisystem organ disorders including polyneuropathy and cardiomyopathy. Amongst the many known pathologic mutations of the transthyretin (*TTR*) gene, the Val122Ile (V122I) mutation can be found in 3–4% of African Americans. Up to 47% of patients with the V122I hATTR cardiomyopathy had a history of carpal tunnel syndrome (CTS). This raises the question should we screen for this mutation in African Americans with bilateral CTS for the purpose of preventing advanced disease associated with hATTR. This is a prospective pilot study to determine the likelihood of African Americans with bilateral CTS having the V122I mutation and whether various clinical factors contribute to that probability.

**Methodology:**

Adult African American patients without prior history of amyloidosis diagnosed with bilateral CTS were recruited for the study. They received genetic testing to screen for a *TTR* mutation. They also completed questionnaires to screen for symptoms of cardiomyopathy and neuropathy, other risk factors for CTS, and family history of CTS and cardiomyopathy.

**Result:**

Two of the sixteen patients (12.5%) in this cohort were found to have the V122I mutation. The absence of polyneuropathy and cardiomyopathy symptoms, presence of other CTS risk factors, and absence of family history of CTS and cardiomyopathy did not decrease the likelihood of V122I mutation in this cohort.

**Conclusion:**

The frequency of V122I transthyretin mutation in African Americans with bilateral CTS may be higher than 3–4%. The presence of bilateral CTS alone may be a justification to screen for *TTR* mutation in this population.

## Introduction

Hereditary transthyretin amyloidosis (hATTR) is an inheritable autosomal dominant disorder. Normal transthyretin (*TTR*) proteins form tetramers that serve as carrier proteins for thyroid hormone and retinol. Mutant *TTR* proteins form unstable tetramers that tend to dissociate into monomers, which can form pathologic amyloid fibrils in various organ systems. The disease manifestations of hATTR include cardiomyopathy, polyneuropathy, autonomic insufficiency, carpal tunnel syndrome (CTS), and more. Many different *TTR* mutations have been described worldwide (1). Among them, the Val30Met mutation is more prevalent in the Portuguese population. It is associated with progressive polyneuropathy more than cardiomyopathy and can have disease manifestation as early as the third decade of life. The Thr60Ala mutation is more prevalent in those with Irish ancestry and often presents with a mixed phenotype of neuropathy and cardiomyopathy. The Val122Ile (V122I) mutation often results in cardiomyopathy, although polyneuropathy can also occur (1).

According to the Transthyretin Amyloid Outcome Survey (THAOS) study, the most prevalent *TTR* mutation in the United States is the V122I mutation. It makes up about 23% of *TTR* amyloidosis cases, with more than 85% of those individuals being African American (2). Previous epidemiology studies, including newborn genetic screening studies, found that 3–4% of the African American population carry at least one copy of the V122I mutation (3–5). When individuals with the V122I mutation develop cardiomyopathy, the diagnosis of hATTR amyloidosis is often not made until the seventh decade, although the onset of cardiac symptoms may precede the diagnosis by a decade (2, 6). The THAOS study also showed that 29.1% of its cohort with V122I hATTR had a history of CTS release surgery (2). Similarly, another retrospective study reported that up to 47% of their cohort with V122I hATTR cardiomyopathy also had CTS (6).

Currently, the presence of a *TTR* mutation with only CTS is not an indication for treatment if there is no evidence of hATTR polyneuropathy or cardiomyopathy. Still, bilateral CTS is one of the so-called Red Flag symptoms that justifies a diagnostic investigation for hATTR, in the appropriate clinical context (7). In addition to peripheral neuropathy and cardiomyopathy, other Red Flag symptoms/signs include autonomic insufficiency, gastrointestinal (GI) symptoms, proteinuria, and vitreous opacity. However, there are no prospective studies examining the predictability of these Red Flag symptoms for hATTR. Whether the presence of bilateral CTS alone justifies screening for hATTR, especially for the purpose of early identification of those at risk for development of cardiomyopathy, remains a topic of interest (8).

Given that the prevalence of the V122I mutation in the African American population is 3–4%, and that up to 47% of these individuals could have had CTS by the time hATTR cardiomyopathy is diagnosed, it raises the question as to whether we should routinely screen for the V122I mutation in African Americans diagnosed with bilateral CTS. Of course, CTS is relatively common, and there can be many other risk factors associated with it. Should we screen for *TTR* mutation in those with bilateral CTS when there are no other risk factors for CTS? Similarly, considering there are other Red Flag symptoms associated with hATTR, should we only screen for *TTR* mutations in those with bilateral CTS in the presence of other Red Flag symptoms? Furthermore, hATTR is an inherited disease, so should a family history of CTS and cardiomyopathy factor into the screening decision as well? Considering that we now have RNA-interference silencing and RNA antisense oligonucleotide drugs for treatment of hATTR neuropathy (9), as well as *TTR* tetramer stabilizer treatment for *TTR* cardiomyopathy (10), it is even more relevant than before, to determine what clinical factors should be used to efficiently identify hATTR before it progresses to advanced disease.

The main objective of this pilot study was to investigate whether the frequency of the V122I mutation in African Americans with bilateral CTS is significantly higher than the rate of 3–4% in the general African American population. In addition, this study explores whether certain Red Flag symptoms, other risk factors for CTS, and family history of CTS or cardiomyopathy affect the probability of this patient population having the V122I mutation.

## Method

### Patient Selection

Patients self-identified as (Black) African American seen in the neuromuscular clinic or EMG lab of University of Florida in Jacksonville, diagnosed with bilateral CTS, were at least 18 years of age, did not have an existing diagnosis of amyloidosis, and could give informed consent were recruited for the study. The study protocol was approved by the University of Florida Institutional Review Board.

### Diagnosis of Carpal Tunnel Syndrome

Patients were given a clinical diagnosis of CTS if they reported intermittent or persistent numbness/tingling in digits 1, 2, or 3 for at least 1 month (11), and had electrophysiological features of median neuropathy at the wrist (12). Those electrophysiological features included significantly prolonged median latency in a mixed transcarpal comparison study between the median and ulnar nerves, significantly prolonged median sensory latency of the palm to digit segment compared with the palm to wrist segment, significantly prolonged median distal latency in the lumbrical-interosseous comparison study, or significantly prolonged median distal motor latency in the appropriate context (i.e., when the aforementioned comparison study responses are unobtainable and without evidence of proximal median motor conduction slowing).

### Genetic Testing

Patients were de-identified prior to their blood or saliva specimen being sent for genetic testing for mutation of the *TTR* gene. Genetic testing was performed through Invitae (sponsored by Alnylam pharmaceuticals), a College of American Pathologists-accredited, Clinical Laboratory Improvement Amendments-certified laboratory. Patients who were found to have a *TTR* mutation were given genetic counseling, referred to appropriate providers, and managed according to current practice standards.

### Questionnaires

Each patient's age, gender, Red Flag symptoms, any risk factors of CTS, and family history of CTS and cardiomyopathy were collected through a set of questionnaires.

### Statistical Analysis

Data was summarized using counts and frequencies (percentages). Associations were assessed using the Fisher's Exact test. The level of significance was set at 5%. All analyses were done in SAS® for Windows Version 9.4.

## Results

Two men and 14 women participated in the study. The average age was 55.75 years, ranging from 38 to 73 years. The results of genetic testing, presence of co-existing Red Flag symptoms, risk factors for CTS, and relevant family history for each patient are presented in Table 1.

**Table 1:** Results of *TTR* mutation screening, with red flag symptom, CTS risk factors, and family history of CTS and cardiomyopathy.

			**Red flag symptoms**				CTS risk factors^*^		**Family history**		
**Patient**	**Age**	**Gender**	**Cardiac symptoms**	**Neuropathy symptoms (sensory)**	**Orthostatic symptoms**	**GI symptoms**	**Diabetes**	**Frequent repetitive tasks**	**Family history of CTS**	**Family history of cardiomyopathy**	***TTR* mutation**
1	63	F	Yes			Yes		Yes			
2	43	F						Yes			
3	72	M						Yes			
4	53	F					Yes	Yes	Yes		
5	61	F	Yes			Yes		Yes	Yes		
6	41	F	Yes		Yes			Yes	Yes		
7	43	F	Yes					Yes	Yes	Yes	
8	63	F	Yes	Yes	Yes	Yes		Yes		Yes	
9	50	F	Yes					Yes	Yes	Yes	
10	38	F				Yes		Yes	Yes		
**11**	**68**	**F**	**Yes**				**Yes**	**Yes**			**V122I (homozygous)**
12	56	M								Yes	
13	41	F	Yes	Yes		Yes		Yes	Yes		
14	65	F	Yes	Yes			Yes	Yes	Yes		
15	73	F	Yes	Yes		Yes		Yes			
**16**	**62**	**F**						**Yes**			**V122I (heterozygous)**

* *Additional CTS risk factors not listed here*.

### Frequency of the V122I Mutation

Two of the 16 patients (12.5%) had the V122I *TTR* mutation. Both were women and in the sixth decade of life. One was heterozygous, and the other was homozygous, for the mutation. The other 14 patients did not have a *TTR* mutation. The percentages of patients with and without TTR mutation, with Red Flag symptoms, other risk factors of CTS, family history of CTS, and cardiomyopathy, are summarized in Table 2.

Table 2Association of red flag symptoms, CTS risk factors, and family history of CTS and cardiomyopathy with V122I *TTR* mutation vs. no mutation.

**Table:** 

	**No *TTR* mutation**	**V122I *TTR* mutation**	***P*-value**
	**14 (87.5%)**	**2 (12.5%)**	
Symptoms of cardiomyopathy	9/14 (64.3%)	1/2 (50%)	0.999
Existing diagnosis of cardiomyopathy	0/14 (0%)	0/2 (0%)	–
Sensory symptoms of neuropathy	4/14 (28.6%)	0/2 (0%)	0.999
Existing diagnosis of neuropathy	3*/14 (21.4%)	1*/2 (50%)	0.450
Symptoms of orthostatic hypotension	2/14 (14.3%)	0/2 (0%)	0.999
GI symptoms	6/14 (42.9%)	0/2 (0%)	0.500
Existing diagnosis of diabetes	2/14 (14.3%)	1/2 (50%)	0.350
Other risk factors for CTS	13/14 (92.8%)	2/2 (100%)	0.999
Family history of cardiomyopathy in 1^st^ degree relative	4/14 (28.6%)	0/2 (0%)	0.999
Family history of CTS in 1^st^ degree relative	8/14 (57.1%)	0/2 (0%)	0.467

**Table:** 

Symptoms of cardiomyopathy	Exertional dyspnea and lower extremity edema
Existing diagnosis of cardiomyopathy	Based on patient reporting
Sensory symptoms of neuropathy	Persistent numbness, burning and tingling in the feet.
Existing diagnosis of neuropathy	Based on patient reporting
Symptoms of orthostatic hypotension	Lightheaded or faint after changing to a more upright position (i.e., from sitting to standing).
GI symptoms	Frequent constipation or diarrhea
Existing diagnosis of diabetes	Based on patient reporting
Other risk factors for CTS	Frequent activities using hands (i.e., typing, cleaning, using power tools, etc.), hypothyroidism, rheumatoid arthritis, monoclonal gammopathy (suspicion of light-chain amyloidosis).

^*^Patients reported a diagnosis of neuropathy but denied typical symptoms of neuropathy.

### Red Flag Symptoms

None of the patients had an existing diagnosis of cardiomyopathy. Symptoms of cardiomyopathy were reported in nine of the patients without the *TTR* mutation. Of the two patients with the V122I mutation, the one with homozygous mutation reported symptoms of cardiomyopathy but had no specific diagnosis.

Three patients without a *TTR* mutation and one of the two patients with V122I mutation reported a prior diagnosis of neuropathy, although they denied the typical sensory symptoms of polyneuropathy.

In those without a *TTR* mutation, two endorsed orthostatic symptoms, and six endorsed GI symptoms. The two patients with the V122I mutation denied orthostatic and GI symptoms.

### Risk Factors and Clinical Features Related to CTS

Two of the patients without a *TTR* mutation and one with the V122I mutation had an existing diagnosis of diabetes. Thirteen of those without a *TTR* mutation and both with the V122I mutation had other risk factors for CTS (mainly frequent repetitive use of the hands). One of the patients with V122I mutation had a diagnosis of rheumatoid arthritis, and the other had a diagnosis of hypothyroidism. Both patients with the V122I mutation endorsed performed jobs or activities that required frequent repetitive use the hands.

### Family History

A family history of cardiomyopathy in one or more first degree relatives was reported in four patients without a *TTR* mutation, and a family history of CTS in one or more first degree relatives was reported in eight patients without a *TTR* mutation. None of the two subjects with the V122I mutation knew of any first-degree relatives with cardiomyopathy or CTS.

## Discussion

### Primary Objective

Our main objective was to examine whether the frequency of the V122I mutation in African Americans who present with bilateral CTS would be greater than the expected 3–4%. We found the V122I mutation in two of the 16 patients (12.5%) who participated in the study. It is interesting to note that both patients with V122I mutation in this cohort were in their 6th decade of life. A previous study showed that for African American patients with V122I hATTR cardiomyopathy, the average age at diagnosis was 70, although the first cardiac symptoms may have preceded the diagnosis by more than a decade (6). In the same study, 47% of those patients had a history of carpal tunnel syndrome. A more recent retrospective study showed for those with *TTR* amyloidosis (including wild-type and hereditary), the probability of having CTS was the highest during the 5–9 years prior to the diagnosis of cardiac amyloidosis, and that CTS was an independent mortality risk factor in transthyretin amyloidosis (13). These findings may further justify screening for the V122I mutation in African Americans who develop carpal tunnel syndrome after their 5th or 6th decade of life, so that those who are found to have the mutation can be evaluated or monitored for cardiomyopathy.

### Explorative Objectives

We also took this opportunity to explore whether some of the Red Flag symptoms, other risk factors for CTS, and family history of CTS and cardiomyopathy would have any predictive value for the V122I mutation in African Americans with bilateral CTS. Prior to the study, it would have been natural to assume that those with co-existing Red Flag symptoms would have an increased likelihood of finding a *TTR* mutation, thus leading one to think it may be low yield to screen for the mutation if there are no Red Flag symptoms. However, in this study, the absence of Red Flag symptoms did not decrease the likelihood of a V122I mutation. Similarly, when patients have risk factors for CTS, it would have been natural to attribute the cause of their CTS to these existing risk factors. However, in this study, the presence of other CTS risk factors did not decrease the likelihood of finding the mutation. Finally, this study showed that the lack of family history of CTS and cardiomyopathy also did not decrease the likelihood of a V122I mutation in this cohort. One must bear in mind that the questionnaires administered in this study regarding Red Flag symptoms, CTS risk factors, and family history were designed to be applicable for real-life clinical settings, where we can ask simple screening questions but are often unable to verify them. Thus, the validity of these findings is contingent on the accuracy of the self-reported symptoms and history provided by the patients.

### Other Findings

This cohort had significantly more women than men. A higher frequency of CTS in women than men has been reported, but whether this difference is due to physiological or occupational risk factors is debated (14, 15). It is uncertain if a patient sample with equal gender representation would have yielded different results.

Of note, two of the patients in the group without a *TTR* mutation developed CTS during pregnancy, and the patients with the V122I mutation did not report that pregnancy was associated with the onset of their CTS symptoms. One cannot not draw the conclusion that CTS onset associated with pregnancy would make it less likely to be related to hATTR, although this may be explored further in a larger study.

### Limitations

This is a small pilot study with major limitations, thus it is uncertain whether these findings would apply to the general African American population. Also, it should be emphasized that all self-reported components of this study (i.e., symptoms onset, co-existing diagnoses, family history, etc.) are subject to recall error, since they were not verified. All patients in this cohort had self-identified as (Black) African American, and from an ethical standpoint, their self-reported racial identity is indisputable. However, from a Mendelian inheritance standpoint, when studying the prevalence of a gene mutation in a racial cohort, individuals of mixed racial origin could affect the carrier frequency of that mutation. The possibility of multi-racial origin in any of the individuals of this cohort was not explored.

The so-called Red Flag symptoms are often found in patients diagnosed with hATTR, and these symptoms (and signs) can justify diagnostic investigations in the clinical setting. However, many of these symptoms are non-specific, and their predictive value for hATTR or *TTR* mutation is unknown. We explored the predictive value of some of the major Red Flag symptom for a *TTR* mutation, such as symptoms of cardiomyopathy, orthostatic hypotension, or GI complaints, and they did not seem to increase the likelihood of a *TTR* mutation in our African American cohort with bilateral CTS. However, the predictive value of other Red Flag symptoms, such as renal dysfunction, vitreous opacities, or unexplained weight loss were not explored.

We did not routinely perform ultrasound studies on patients with CTS. However, a recent study using peripheral nerve ultrasound showed the average median nerve cross-sectional area was smaller in those with *TTR* mutation than those with idiopathic CTS. In that study, increased median nerve cross-sectional area correlated with increased severity of idiopathic CTS, but this correlation was not apparent in those with *TTR* mutation (16). Whether ultrasound features can serve as a predictive factor for the likelihood of having *TTR* mutation in patients with CTS remains an interesting area of exploration.

It must be emphasized that those with the V122I mutation in this study were not confirmed to have amyloidosis. Confirmation of hATTR causing CTS would require tenosynovial biopsy during CTS surgery, which was not a part of this study. It is also possible to have a false negative biopsy in an individual with amyloidosis simply due to sampling error. Not every case of CTS requires surgery and carpal tunnel release surgery is often performed for more severe cases or those refractory to non-surgical management. A nationwide Danish registry study that included 56,032 patients showed CTS surgery was associated with higher risk of amyloidosis and heart failure compared to control subjects, although this study did not distinguish the type of amyloidosis (17). In a prospective study examining men ages ≥50 and women ages ≥60 undergoing CTS surgery (85% of those with bilateral CTS), 10 out of the 98 patients (10.2%) were found to have amyloid deposits, although only seven had TTR amyloid deposition and only two of those patients had TTR mutations (Leu58His and Ala81Thr) (18). In comparison, our prospective study screening African American patients with bilateral CTS identified two out of 16 (12.5%) patients with V122I *TTR* mutation. If this probability is reproducible in a larger study, it could be clinically impactful. Biopsy confirmation of ATTR deposition in patients with bilateral carpal tunnel syndrome with V122I *TTR* mutation may be possible in a future study with long term follow up as well.

Ultimately, whether identification of the V122I mutation based on CTS prior to the onset of cardiomyopathy, polyneuropathy, or dysautonomia will ultimately impact mortality and morbidity associated with hATTR, remains to be determined.

### Justification for Future Studies

This was a prospective evaluation of the predictability of bilateral CTS for the V122I mutation in an African American population, and we found that 12.5% of our cohort had the mutation. We focused on the African American population, because carrier rate of the V122I mutation in this population was already established to be 3–4%, which serves as a benchmark, in that any screening approach that identifies a higher percentage of the mutation can be clinically impactful. The greater purpose of this effort is to determine efficient ways to identify *TTR* mutations with the aim of preventing advanced disease associated with hATTR. Despite the limitations of this study, the findings here could suggest that the presence of bilateral CTS in African American individuals, especially in their 5th or 6th decade of life, may be an adequate justification to screen for a *TTR* mutation, regardless of Red Flag symptoms, other risk factors for CTS, or family history of CTS or cardiomyopathy. Of course, a larger study would help to verify these findings. Ideally, a future study would address the limitations of this study. Furthermore, in individuals found to have a *TTR* mutation on the basis of bilateral CTS, it would be valuable to determine what proportion of them may already have cardiomyopathy. Ultimately, if screening for a *TTR* mutation based on a diagnosis of bilateral CTS leads to identification of otherwise overlooked hATTR cardiomyopathy or prevention of advanced hATTR-associated disease in a significant number of African American individuals, it may be justified to incorporate this screening approach into a practice guideline.

## Data Availability Statement

The original contributions presented in the study are included in the article/Supplementary Material, further inquiries can be directed to the corresponding author/s.

## Ethics Statement

The studies involving human participants were reviewed and approved by University of Florida, Jacksonville, Florida, Institutional Review Board. The patients/participants provided their written informed consent to participate in this study.

## Author Contributions

JS designed and carried out the study, wrote the abstract, introduction, much of the methodology, results and discussion of the manuscript. MB contributed to the design of a database for the study, carried out the study, and contributed to the methodology section of the manuscript. CS contributed to the design of the database, performed statistical analysis, and contributed to the methodology section of the manuscript. All authors contributed to the article and approved the submitted version.

## Funding

University of Florida internal funding.

## Conflict of Interest

JS has participated on the Alnylam Pharmaceuticals advisory board in 2020, given a disease awareness talk in 2021 for Alnylam, and received financial compensations for those activities. The remaining authors declare that the research was conducted in the absence of any commercial or financial relationships that could be construed as a potential conflict of interest.

## Publisher's Note

All claims expressed in this article are solely those of the authors and do not necessarily represent those of their affiliated organizations, or those of the publisher, the editors and the reviewers. Any product that may be evaluated in this article, or claim that may be made by its manufacturer, is not guaranteed or endorsed by the publisher.

## Abbreviations

CTS: carpal tunnel syndrome
hATTR: hereditary transthyretin amyloidosis
THAOS: transthyretin amyloid outcome survey
TTR: transthyretin
V122I: Val122Ile.
